# A custom next generation sequencing panel to identify the cause of monogenic disorders of insulin secretion, disorders of sexual development and noonan syndrome

**DOI:** 10.1186/1687-9856-2015-S1-P87

**Published:** 2015-04-28

**Authors:** Ivan McGown, Mark Williams, Sam McManus, Tony Huynh, James Harraway

**Affiliations:** 1 Molecular Genetics Department, Mater Pathology, Mater Health Services, South Brisbane, QLD, Australia; 2 Clinical Chemistry Department, Mater Pathology, Mater Health Services, South Brisbane, QLD, Australia

## 

Next generation sequencing (NGS), using massive parallel sequencing, is increasingly being used in the clinical setting. It is a high throughput technique that offers a fast and cost-effective testing solution for genetic disorders with a number of candidate genes.

In this study we have employed NGS to sequence 48 samples that had been referred to the Molecular Genetics Department for the investigation of Monogenic Disorders of Insulin Secretion (MDOIS), Disorders of Sexual Development (DSD) and Noonan Syndrome (NS). All samples had previously been genotyped using Sanger sequencing.

Next Generation Sequencing was performed on an Illumina MiSeq using an Illumina Nextera Rapid Capture Custom Enrichment Kit. This custom assay contained capture probes for the coding regions (including +/- 5 bases of intronic sequence) for 66 genes: 34 for DSD; 19 for MDOIS; and 13 for NS. Sequence data generated by the MiSeq was aligned to hg19 and variants detected using CLC Genomics Workbench. Genetic variants were annotated using Cartagenia BENCHlab NGS.

Thirty mutations associated with DSD (SRD5A2, HSD17B3, NR5A1, AR), MDOIS (ABCC8, GCK, GLUD1, INS, HNF1A, HNF4A, HNF1B) and NS (PTPN11, RAF1) were detected. This was concordant with genotypes detected by Sanger sequencing.

Next generation sequencing has proven to be an accurate and efficient method of genotyping monogenic disorders with multiple candidate genes, and is an ideal technique for the clinical laboratory.

